# miR-877-5p Inhibits Epithelial Mesenchymal Transformation of Breast Cancer Cells by Targeting FGB

**DOI:** 10.1155/2022/4882375

**Published:** 2022-11-17

**Authors:** Haixia Liu, Lili Xiang, Yu Mei

**Affiliations:** ^1^Department of Pathology, Jinan Maternity and Child Care Hospital, 250001 Jinan, Shandong, China; ^2^Department of Children's Health Care, Jinan Maternity and Child Care Hospital, 250001 Jinan, Shandong, China; ^3^Department of Breast Surgery, Jinan Maternity and Child Care Hospital, 250001 Jinan, Shandong, China

## Abstract

**Purpose:**

This present study is aimed at exploring the FGB expression in breast cancer (BC) and the role of FGB in BC.

**Methods:**

A total of 150 pairs of BC tissues and adjacent tissues from BC surgery patients were collected. RT-qPCR was utilized to evaluate the mRNA expression of FGB and miR-877-5p. Immunohistochemistry was applied to evaluate the protein expression of FGB. Chi-square test was performed to evaluate the relationship between FGB expression level and clinical characteristics. Cell proliferation was examined using CCK-8 assay. Cell invasion was evaluated by transwell assay. Flow cytometry assay was applied to measure cell apoptosis. The protein expression was evaluated by western blot. BALB/C nude mice were used to establish the xenograft tumor model.

**Results:**

FGB was more highly expressed in BC tumor, and the expression of FGB was relevant to TNM stage and lymph node metastasis and showed a positive correlation. FGB was proved to be directly regulated via miR-877-5p and enhanced proliferation and invasion of BC cells. FGB downregulation markedly inhibited the tumor growth, including tumor weight and volume. In addition, the Ki-67 expression was observably declined in the sh-FGB group. The protein expression of E-cadherin was markedly raised in the sh-FGB group while the protein expression of N-cadherin and vimentin was markedly declined in the sh-FGB group.

**Conclusion:**

In conclusion, miR-877-5p inhibits epithelial mesenchymal transformation, cell proliferation, and invasion of BC cells via downregulating FGB.

## 1. Introduction

BC is a common fatal malignancy, with morbidity and mortality ranking at the forefront of female malignancies. There are about 1.7 million new cases worldwide each year and about 170,000 in China [1]. With the application of various therapies such as surgery, radiotherapy, and endocrine drugs, the prognosis and survival of patients have been improved [2]. Inhibition of tumor cell invasion and metastasis is an urgent scientific problem. Many BC patients have metastases before diagnosis, which will weaken the treatment effect and have a poor prognosis [3]. Hence, it is important to investigate molecular mechanism of BC occurrence, invasion, and metastasis and explore clinical therapeutic targets for early diagnosis and treatment of BC.

MicroRNAs (miRNAs) are a class of endogenous noncoding single-stranded RNA molecules of 18-24 nucleotides in length [4]. The miRNAs suppress the target protein expression or promote mRNA degradation by complementary binding to the 3′UTR of mRNA [5]. Currently, miRNA is found to play a role in various physiological and pathological processes of numerous tumors, containing cell cycle, proliferation, invasion, metastasis, and other biological functions [5]. Multiple specific miRNAs are abnormally expressed in BC tumor tissues, suggesting their involvement in process of BC progression [6, 7]. The mechanisms of miRNA involvement in tumor invasion and metastasis are complex, but epithelial-mesenchymal transition (EMT) has been widely studied in cancer and is believed to be essential in tumor deterioration, which is regulated by a variety of miRNAs [8]. For example, miR-520c-3p negatively regulated EMT to inhibit cell invasion and migration in BC by targeting IL-8 [7]. miR-130a-3p was poorly expressed in BC cell, and the overexpression of miR-130a-3p impeded BC cell migration and invasion [6]. The involvement of in EMT refers to the process by which tumor cells of epithelial origin lose their epithelial phenotype and acquire a mesenchymal phenotype, thereby gaining the ability to invade and metastasize. EMT is the first and crucial step for tumor cells to undergo invasive metastasis. Fibrinogen beta chain (FGB) was reported to be more obviously highly expressed in the malignant pulmonary nodules by comparison with the benign pulmonary nodules [9]. However, the expression of FGB in BC and whether FGB could regulate the EMT process in BC by miRNAs currently remains unclear.

This present study is aimed at measuring the expression of FGB in BC and the effect of FGB deficiency on progress of BC and EMT.

## 2. Methods

### 2.1. Tissue Samples

A total of 150 pairs of BC tissue and adjacent tissue from BC surgery invalids admitted to the hospital from May 2018 to December 2020 were taken. All the patients gave their informed consent. Patients who received radiotherapy or chemotherapy or other anticancer treatment before operation were excluded. After tissue samples was removed, they were stored at -80°C for use. The tumors of the patients were immunostained for ER (SP1 antibody), PR (1E2 antibody), and HER2 (4B5 antibody).

### 2.2. Cell Culture and Transfection

BC cell MCF-7 was purchased from American Type Culture Collection (ATCC) and maintained in DMEM. 10% fetal bovine serum, 100 U/ml streptomycin, and 100 U/ml penicillin were added to the medium. Cell culture environment was 5% CO_2_ and 37°C. MCF-7 cells were transfected using sh-FGB or sh-NC by Lipofectamine 2000 reagent.

### 2.3. RT-qPCR Assay

Total RNA was extracted by the TRIzol method (Absin, China). cDNA was obtained according to the instructions of reverse transcription kit (Absin, China). The amplification of the target gene was detected by SYBR Premix Ex Taq (Takara, Japan).  Table 1 shows the primer sequences.

### 2.4. Western Blot

MCF-7 cells transfected for 48 h were collected, total protein was extracted, and protein concentration was detected via BCA. After the SDS-PAGE separation, protein was transferred into the PVDF membrane. PVDF membrane was blocked using 5% skim milk powder solution at 4°C overnight. The membrane was incubated using FGB, Ki-67, E-cadherin, vimentin, N-cadherin, or *β*-actin antibodies for 2 h at 25°C. The membrane was incubated using secondary antibodies for 1 h. Chemiluminescence was developed using a hypersensitive ECL kit (Baiaosi, China).

### 2.5. CCK-8 Assay

MCF-7 cells transfected using si-FGB and negative control were cultured for one day and digested using 0.25% trypsin. Cell suspension was diluted at 5 × 10^5^ pieces/ml. 10 *μ*L cell suspension was inoculated into 96-well plate. Cells were incubated with 10 *μ*L CCK-8 solution at 37°C for 1 h, and then the absorbance of 450 nm was detected by microplate reader.

### 2.6. Transwell Assay

The transwell chamber was placed in a 24-well plate. After evenly mixing the Matrigel matrix gel with DMEM in a 1 : 6 ratio, 50 *μ*L was added to the bottom of the transwell chamber. Complete medium including FBS was added to the down chamber, and 1 × 10^5^ cells in 200 *μ*L medium were added to the upper chamber for 24 h. Cell number attached to the lower surface of the compartment was observed using a microscope.

### 2.7. Luciferase Reporter Assay

The fragment of miR-877-5p bound to FGB was amplified and inserted into the luciferase reporter plasmid to construct the wild-type FGB plasmid (FGB-WT). The mutant FGB plasmid (FGB-MUT) was constructed after the binding site. Subsequently, 293 T cells were transfected with FGB-WT and FGB-MUT with miR-877-5p mimics, and the fluorescence intensity of each group was measured after 48 h using the Luciferase Reporter Assay Kit.

### 2.8. Flow Cytometry Assay

Cells were suspended in 500 *μ*L Binding Buffer. The cell suspension was added with 5 *μ*L Annexin V-FITC and stained for 15 min at 25°C. 5 *μ*L PI dye was added and stained for 5 min at 25°C. Flow cytometry was utilized to measure apoptosis.

### 2.9. Xenograft Tumor Model in BALB/C Nude Mice

BALB/c nude mice were injected with si-NC or si-FGB under the skin of the left flank. Tumor growth was observed on days 9, 14, 19, 24, and 29. On the 29th day, all mice were sacrificed, and tumor tissues were taken out and weighed.

### 2.10. Statistical Analysis

Analysis was conducted via SPSS 25.0 software. Comparison between two groups was performed by *t*-test and one-way ANOVA between multiple groups. *P* < 0.05 represented that differences were statistically significant.

## 3. Results

### 3.1. FGB Was More Highly Expressed in BC Tissues

**Figure 1:**
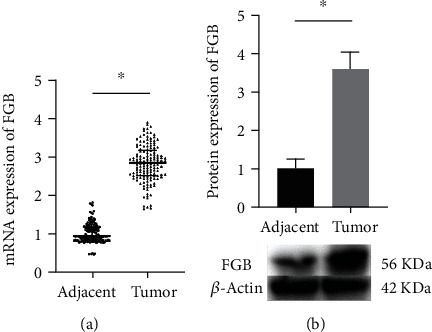
FGB was upregulated in BC tissues. (a) The mRNA expression of FGB was evaluated by qRT-PCR. (b) The expression of FGB was measured in by western blot. ^∗^*P* < 0.05.

The mRNA expression of FGB was obviously raised in BC tumor tissues (Figure 1(a)). By comparison with adjacent tissues, FGB was more highly expressed in BC tumor tissues (Figure 1(b)). The expression of FGB was associated with TNM stage and lymph node metastasis and showed a positive correlation (Table 2). The expression of FGB was not obviously correlated with other indicators.

### 3.2. Knockdown of FGB Suppressed the Proliferation and Invasion of BC Cells

**Figure 2:**
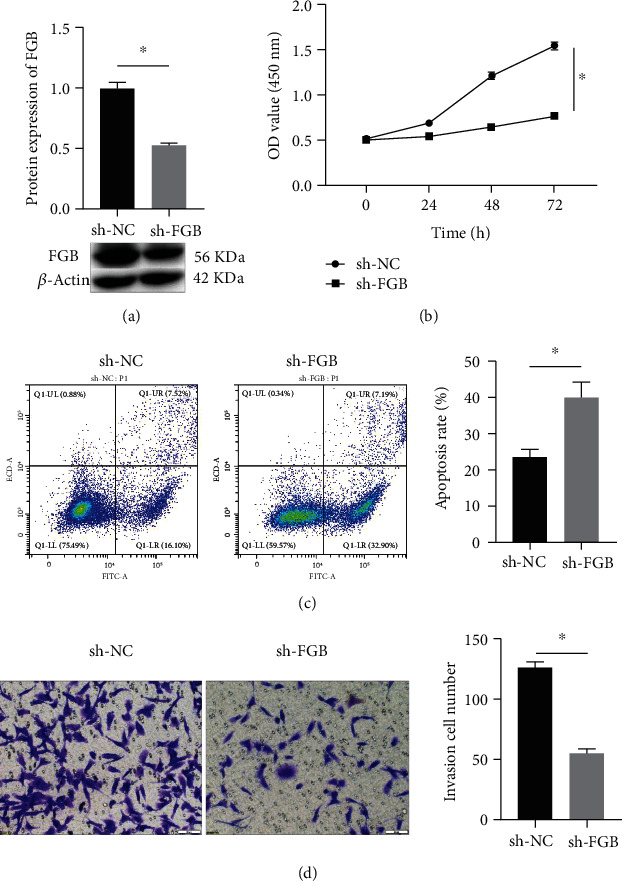
Knockdown of FGB suppressed the proliferation and invasion of BC cells. (a) Western blot was utilized to detect the protein expression of FGB. (b) Hindrance effect of FGB knockdown on MCF-7 cell proliferation. (c) Hindrance effect of FGB knockdown on MCF-7 cell apoptosis. (d) Hindrance effect of FGB knockdown on MCF-7 cell invasion. Magnification ×200.

Western blot was utilized to evaluate the knockdown effect and demonstrate the expression of FGB which was obviously declined in the sh-FGB group (Figure 2(a)). Cell proliferation ability was inhibited by sh-FGB in contrast to the sh-NC group (Figure 2(b)). Knockdown of FGB obviously promoted BC cell apoptosis in contrast to the sh-NC group (Figure 2(c)). Knockdown of FGB markedly decreased BC cell invasion in contrast to the sh-NC group (Figure 2(d)).

### 3.3. Knockdown of FGB Suppressed the Progress of BC In Vivo

**Figure 3:**
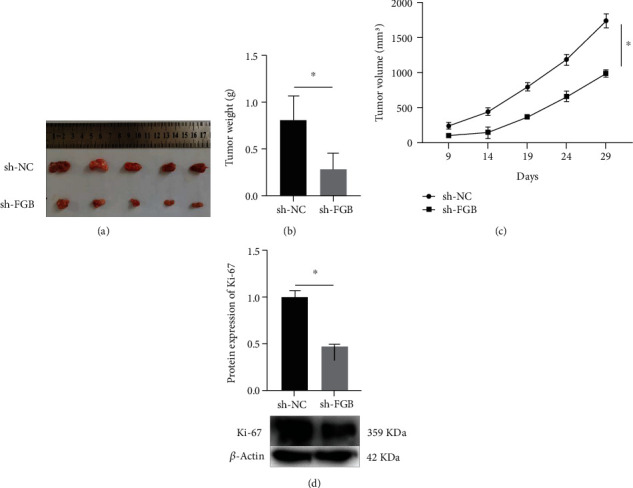
Knockdown of FGB suppressed the progress of BC in vivo. (a) Tumor photos taken by digital camera. (b) Tumor weight was obviously declined in the sh-FGB group. (c) Tumor volume was markedly declined in the sh-FGB group. (d) The Ki-67 protein expression was evaluated by western blot. ^∗^*P* < 0.05.

The xenograft tumor model was constructed to figure out the influence of FGB knockdown on tumor growth in vivo. As shown in Figure 3(a), knockdown of FGB markedly inhibited the tumor growth. In contrast to the sh-NC group, knockdown of FGB markedly decreased tumor weight (Figure 3(b)) and volume (Figure 3(c)). The Ki-67 expression was observably declined in the sh-FGB group (Figure 3(d)).

### 3.4. FGB Was Directly Regulated by miR-877-5p

**Figure 4:**
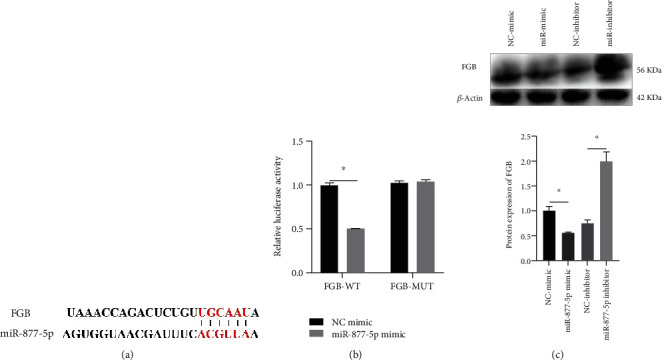
FGB was directly regulated by miR-877-5p. (a) Predicted binding sites. (b) Luciferase reporter assay was conducted. (c) The protein expression of FGB was measured via western blot. ^∗^*P* < 0.05.

TargetScan online tool was utilized to predict the upstream of FGB and found that FGB might be regulated by miR-877-5p (Figure 4(a)). Luciferase reporter assay confirmed this prediction. miR-877-5p mimic obviously decreased the relative luciferase activity of FGB-WT while the relative luciferase activity of FGB-MUT was not changed (Figure 4(b)). The FGB expression was inhibited in the miR-877-5p mimic group while the FGB expression was raised in the miR-877-5p inhibitor group (Figure 4(c)).

### 3.5. Knockdown of FGB Suppressed the EMT Progress of BC

**Figure 5:**
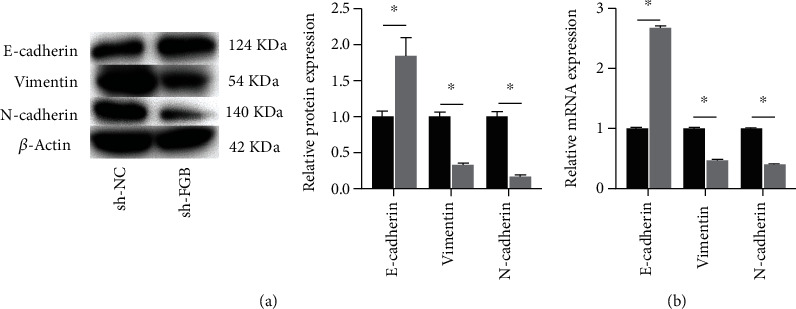
Knockdown of FGB suppressed the EMT progress of BC. (a) Western blot was utilized to measure the protein expression. (b) RT-qPCR was utilized to measure the mRNA expression. ^∗^*P* < 0.05.

The protein expression of E-cadherin was markedly raised in the sh-FGB group while the protein expression of N-cadherin and vimentin was observably decreased in the sh-FGB group (Figure 5(a)). The mRNA expression of E-cadherin was observably raised in the sh-FGB group while the mRNA expression of N-cadherin and vimentin was markedly declined in the sh-FGB group (Figure 5(b)).

## 4. Discussion

Local recurrence or distant metastasis may occur after BC surgery. Cancer cells can metastasize because of uncontrolled cell proliferation and migration [10]. Once the free cancer cells spread to the whole body and form cancer metastasis, they will be life-threatening. In recent years, a large number of studies have reported that miRNAs were essential in tumor invasion and metastasis in BC [11]. The present study found that FGB was more highly expressed in BC tumor tissue, and the expression of FGB was relevant to TNM stage and LNM and showed a positive correlation. In addition, FGB was directly regulated by miR-877-5p and enhanced the proliferation and invasion of BC cells. miRNAs were essential in progress of tumor development and EMT, which could regulate the process of tumor EMT mediated by oncogenes or tumor suppressor genes, so as to regulate the process of tumor development [12]. miR-646 inhibited EMT-induced proliferation and metastasis by targeting FOXK1 [13]. miR-1249 inhibited EMT progression, proliferation, and migration in BC cells [14]. In gastric cancer cells, miR-130a-3p inhibited cell migration and invasion via inhibiting the TBL1XR1-mediated EMT process [15]. Inhibition of miR-877-5p promoted migration and invasion of gastric cancer cells via binding to FOXM1 [16]. miR-877-5p inhibited cell proliferation in prostate cancer via binding SSFA2 [17].

In order to figure out the influence of FGB knockdown on tumor growth in vivo, the xenograft tumor model was constructed successfully. Results showed that knockdown of FGB markedly inhibited the tumor growth, including tumor weight and volume. In addition, the Ki-67 expression was observably declined in the sh-FGB group. These results demonstrated that miR-877-5p could inhibit the BC progress by targeting FGB in vivo. It has been proved that EMT can regulate the invasion and migration of tumor cells [18, 19]. EMT is mainly characterized by dissociation of epithelial tight junctions, loss of cellular adhesions, and cytoskeletal rearrangements [20]. The main hallmarks of EMT are a decrease in epithelial cellular markers (E-cadherin) and an increase in mesenchymal cellular markers (N-cadherin, vimentin). E-cadherin, N-cadherin, and vimentin also became the main markers for the evaluation of EMT [20]. The present study demonstrated that the protein expression of E-cadherin was obviously raised in the sh-FGB group while the protein expression of N-cadherin and vimentin was markedly decreased in the sh-FGB group. Therefore, FGB could promote the EMT process to enhance deterioration degree of BC.

In conclusion, miR-877-5p inhibits epithelial mesenchymal transformation, cell proliferation, and invasion of BC cells by targeting FGB.

## Figures and Tables

**Table 1:** Primer sequence.

Target	Primer sequence (5′-3′)
miR-877-5p	F: TAGAGGAGATGGCGCAG
	R: GAACATGTCTGCGTATCTC

U6	F: CTCGCTTCGGCAGCACA
	R: AACGCTTCACGAATTTGCGT

FGB	F: AGCCTACAGATCACTAGCAAT
	R: TGTGGTACTGATGCTCTCCACG

*β*-Actin	F: ACGTCACGAACTACTAGCAAT
	R: TGTGTGCATGAGTCTCTCCACG

**Table 2:** Relationship between the expression of FGB and clinical features.

Items	*N*	Low (*n* = 76)	High (*n* = 74)	*χ* ^2^	*P*
Age, year				0.496	0.481
≤50	59	32	27		
>50	91	44	47		
Tumor size, cm				3.311	0.069
≤3	68	40	28		
>3	82	36	46		
ER status				1.102	0.294
Negative	57	32	25		
Positive	93	44	49		
PR status				1.354	0.245
Negative	82	38	44		
Positive	68	38	30		
HER2 status				1.312	0.252
Negative	76	35	41		
Positive	74	41	33		
TNM stage				6.014	0.014
I/II	86	51	35		
III/IV	64	25	39		
Lymph nodes status				5.311	0.021
Negative	91	53	38		
Positive	59	23	36		

## Data Availability

Data to support the findings of this study is available on reasonable request from the corresponding author.
